# A case study on the genetic origin of the high oleic acid trait through *FAD2-1* DNA sequence variation in safflower (*Carthamus tinctorius* L.)

**DOI:** 10.3389/fpls.2015.00691

**Published:** 2015-09-09

**Authors:** Sara Rapson, Man Wu, Shoko Okada, Alpana Das, Pushkar Shrestha, Xue-Rong Zhou, Craig Wood, Allan Green, Surinder Singh, Qing Liu

**Affiliations:** ^1^Commonwealth Scientific and Industrial Research Organization AgricultureCanberra, ACT, Australia; ^2^State Key Laboratory of Cotton Biology, Institute of Cotton Research of Chinese Academy of Agricultural SciencesAnyang, China; ^3^Commonwealth Scientific and Industrial Research Organization Land and WaterCanberra, ACT, Australia; ^4^Indian Council of Agricultural Research, Central Plantation Crops Research InstituteRC, Assam, India; ^5^Commonwealth Scientific and Industrial Research Organization Food and NutritionCanberra, ACT, Australia

**Keywords:** hybridization, integration, phylogenetics, safflower, *FAD2-1*

## Abstract

The safflower (*Carthamus tinctorius* L.) is considered a strongly domesticated species with a long history of cultivation. The hybridization of safflower with its wild relatives has played an important role in the evolution of cultivars and is of particular interest with regards to their production of high quality edible oils. Original safflower varieties were all rich in linoleic acid, while varieties rich in oleic acid have risen to prominence in recent decades. The high oleic acid trait is controlled by a partially recessive allele *ol* at a single locus OL. The *ol* allele was found to be a defective microsomal oleate desaturase *FAD2-1*. Here we present DNA sequence data and Southern blot analysis suggesting that there has been an ancient hybridization and introgression of the *FAD2-1* gene into *C. tinctorius* from its wild relative *C. palaestinus*. It is from this gene that *FAD2-1*Δ was derived more recently. Identification and characterization of the genetic origin and diversity of *FAD2-1* could aid safflower breeders in reducing population size and generations required for the development of new high oleic acid varieties by using perfect molecular marker-assisted selection.

## Introduction

Hybridization between domestic crop plants and their wild relatives, and the potential for gene introgression, has received widespread interest in recent years (Ellstrand et al., 1999; Jarvis and Hodgkin, 1999; Felber et al., 2007; Arrigo et al., 2011). Spontaneous hybridization between domesticated and wild plants generally occurs wherever the crop plants remain in their place of origin. Genetic introgression has been defined as “the permanent incorporation of genes from one set of differentiated populations into another” (Stewart et al., 2003), and the gene introgression from wild relatives has long been considered important for the evolution of domestic crop species (Stebbins, 1959; Harlan, 1965; Slatkin, 1987; Prescott-Allen and Prescott-Allen, 1988; van Raamsdonk and van der Maesen, 1996). In these cases, the maintenance of new genetic combinations, which result in populations with new characteristics, depends on both natural and human selection (Jarvis and Hodgkin, 1999). There are many well-known breeding programmes involving deliberate introgression of desirable traits into crop plants as well as a growing number of studies documenting the natural hybridization and gene introgression between agricultural crops and their wild relatives (reviewed by Jarvis and Hodgkin, 1999). This is of particular interest in safflower (*Carthamus tinctorius* L.) with regards to high quality oil production.

*C. tinctorius* is considered a strongly domesticated species with a long history of cultivation and widespread distribution across the globe (Dempewolf et al., 2008). Hybridization with several sympatric wild species of *Carthamus* in the Mediterranean and Asia is suggested to have played an important role in the evolution of *C. tinctorius* (Ashri and Knowles, 1960; Schank and Knowles, 1964; Vilatersana et al., 2007). Indeed, hybridization between *C. tinctorius* and several wild relatives has been demonstrated to occur both artificially by hand pollination (Heaton and Klisiewicz, 1981), and naturally by open pollination (Ashri and Rudich, 1965). Notably, *C. tinctorius* can readily cross with both *C. oxyacanthus* and *C. palaestinus* to produce fertile hybrids (McPherson et al., 2004; Mayerhofer et al., 2011). Given that all three species coexist in the Old World and the range of *C. oxyacanthus* in the New World overlaps with the cultivated *C. tinctorius*, there is clear biological potential of an interspecific hybridization event should they be temporally sympatric. Each with 12 chromosomes, these species can be easily crossed, giving rise to fertile hybrids (Gill et al., 2011).

Safflower is grown mainly for its oil that can be classified into two types, one being characterized by high level of linoleic acid and the other being rich in oleic acid (HO varieties). While the oil with high level of linoleic acid is regarded as one of the most highly polyunsaturated vegetable oils (Velasco and Fernandez-Martinez, 2001), the oil with high level of oleic acid is considered particularly valuable because, in addition to its cholesterol-lowering effect, the high oleic oil also has high oxidative stability. These traits make it ideal for food applications without partial hydrogenation that can generate nutritionally undesirable *trans* fatty acids (Kinney and Clemente, 2005). The original safflower varieties were all high linoleic type. The high oleic trait that is controlled by a partially recessive allele *ol* at a single locus OL was first identified in an introduction from India (Knowles and Bill, 1964). The *ol* allele was subsequently incorporated into safflower breeding programs and the first HO safflower variety “UC-1” was released in 1966 in the US, which was followed by the releases of “Oleic leed” and the Saffola series including Saffola 317 (S-317), S-517, and S-518. The *ol* allele has also been used as the background genetic material for further enhancement of oleic acid content in safflower breeding programs worldwide (Weiske, 1997; Mundel and Bergman, 2009).

Oleic acid level in plant seed oil is primarily determined by the activity of microsomal Δ12 oleate desaturase FAD2 (Okuley et al., 1994). We isolated an unusually large *FAD2* gene family with 11 members from safflower (Cao et al., 2013), among which *FAD2-1* was demonstrated to be the key oleate desaturase gene specifically expressed in developing seeds and plays the major role in producing linoleic acid in safflower oil, while *FAD2-2* appears to encode the house-keeping microsomal Δ12 oleate desaturase that has a generally constitutive expression throughout the plant. Other members of the *FAD2* gene family were found to have divergent gene functions, with little role, if any, in the fatty acid composition of safflower seed oil (Cao et al., 2013). The *ol* allele in the high oleic variety was found to be a defective *FAD2-1* with a single nucleotide deletion in the coding region that leads to premature termination of translation and subsequent nonsense-mediated mRNA decay (NMD) of *FAD2-1*, a process that typically degrades transcripts containing a premature termination codon (PTC) (Guan et al., 2012; Liu et al., 2013).

Resolving evolutionary questions within *Carthamus* has been challenging as low levels of genetic variation belie clear morphological differences between species (Vilatersana et al., 2005; Bowles et al., 2008). The development of molecular genetic techniques has greatly facilitated our ability to study low levels of introgression. In addition to the expressed-sequence tag (EST) and simple sequence repeat (SSR) markers used by Chapman et al. (2009), random amplified polymorphic DNA markers (Vilatersana et al., 2005), and conserved intron-spanning PCR markers (Chapman and Burke, 2007) have also been employed in studies addressing species relationships.

In this paper we present DNA sequence data and Southern blot analysis suggesting that there has been hybridization and introgression of the *FAD2-1* gene from *C. palaestinus* into *C. tinctorius*, from which the *ol* allele (*FAD2-1*Δ) was derived more recently. We have mainly focused on the use of a large intron (~1.2 kb) present in the *FAD2-1* 5′ untranslated region (5′ UTR) which may evolve with less, if any, functional constrains and is therefore more suitable for elucidating evolutionary history than a coding region (Small et al., 2004). Such a 5′ UTR intron in *FAD2-1* was previously used in evolutionary studies of *Gossypium* species (Liu et al., 2001). It has been suggested that *FAD2-1* intron is large enough to be evolutionary meaningful, and it may be evolving at a quick enough rate for inferring evolutionary relationships among recently diverged lineages (Liu et al., 2001). In this regard it is particularly useful for elucidating the evolutionary pathways of *Carthamus* species.

## Materials and methods

### Plant materials and genetic extraction

In this study we used 16 *C. tinctorius* accessions (populations), 5 accessions of *C. oxycanthus*, and 1 accession of *C. palaestinus*, all of which were obtained from USDA Western Regional Plant Introduction Station (WRPIS; http://www.ars.usda.gov/main/site_main.htm?modecode=53481500; Table 1).

**Table 1:** **Specimen details, their corresponding symbols, and GenBank numbers assigned to relevant DNA sequences**.

**USDA Germplasm Accession**	**Symbol**	**Plant ID**	**Country of Origin**	**Species**	**GenBank Accession No. of *FAD2-1***	**GenBank Accession No. of *FAD2-2***
PI 209295	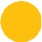	BJ-781	Kenya	*C. tinctorius*	KT193562	KT193585
PI 239042	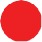	BJ-820	Morocco	*C. tinctorius*	KT193563	KT193586
PI 250606	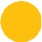	BJ-1067	Egypt	*C. tinctorius*	KT193564	KT193587
PI 253759	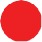	BJ-1213	Iraq	*C. tinctorius*	KT193565	KT193588
PI 262433	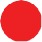	BJ-2716	Ethiopia	*C. tinctorius*	KT193566	KT193589
PI 271070	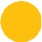	TOZI SPINY	Sudan, Northern	*C. tinctorius*	KT193567	KT193590
PI 279051	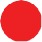	U. California 61-20	India	*C. tinctorius*	KT193568	KT193591
PI 292000	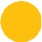	ELS 6404-63-2	Israel	*C. tinctorius*	KT193569	KT193592
PI 301053	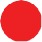	Kayit 5-65	Turkey	*C. tinctorius*	KT193570	KT193593
PI 401472	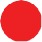	BJ-2025	Bangladesh	*C. tinctorius*	KT193571	KT193594
PI 401479	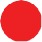	BJ-2032	Bangladesh	*C. tinctorius*	KT193572	KT193595
PI 544041	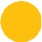	Honghua	China, Xizang	*C. tinctorius*	KT193573	KT193596
PI 576992	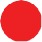	CART 72/86	Korea, North	*C. tinctorius*	KT193574	KT193597
PI 613459	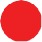	80/131/BS	Portugal	*C. tinctorius*	KT193575	KT193598
PI 599253	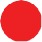	S-317	Cultivar	*C. tinctorius*	KT193576	KT193599
PI 401577	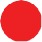	NP12	Cultivar	*C. tinctorius*	KT193577	KT193600
PI 538779	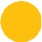	Centennial	Cultivar	*C. tinctorius*	KT193578	–
PI 235663	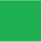	BJ-1964	Israel	*C. palaestinus*	KT193579	KT193601
PI 426185	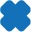	K-1076	Afghanistan	*C. oxyacantha*	KT193580	KT193602
PI 426428	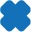	K-2	Pakistan	*C. oxyacantha*	KT193581	KT193603
PI 426443	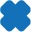	K-401	Pakistan	*C. oxyacantha*	KT193582	KT193604
PI 426447	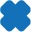	K-414	Pakistan	*C. oxyacantha*	KT193583	KT193605
PI 426488	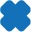	K-753	Pakistan	*C. oxyacantha*	KT193584	KT193606

### Genetic sequencing

The genomic DNA of *Carthamus* entries was isolated from fully expanded young leaves using CTAB buffer and further purified by CsCl gradient as described by Cao et al. (2013). The intron located in the 5′ UTR of *FAD2-1* was amplified using the following oligonucleotides: 5′- GAGATTTTCAGAGAGCAAGCGCTT -3′ and 5′- CTTTGGTCTCGGAGGCAGACATA -3′ for *FAD2-1* and 5′- CAAAAGGAGTTTCAGAAAGCCTCC -3′ and 5′- ACTCGTTGGATGCCTTCGAGTTC- 3′ for *FAD2-2*. All polymerase chain reaction (PCR) amplification reactions were performed in a final volume of 20 μL using 10 μL HotStart PCR mix (Qiagen, Hilden, Germany), 0.3 μM of each forward and reverse primers and 20 ng of *Carthamus* genomic DNA template. Reaction mixtures were denatured at 95°C for 15 min before undergoing 35 amplification cycles (94°C for 30 s, 57°C for 30 s and 72°C for 1 min), followed by a final extension at 72°C for 10 min. PCR products were subsequently purified with QIAquick® PCR Purification Kit (QIAGEN), and cloned into pGEM-T easy® (Promega, Madison, WI, USA). The entire inserts were sequenced bidirectionally using Big Dye Terminator (Applied Biosystems, Melbourne, Australia) reactions and reaction products separated on a capillary sequencer (Applied Biosystems). Sequences were assembled using CHROMAS PRO (Technelysium Ltd, Brisbane, Australia) and have been deposited in GenBank (Accession numbers are listed in Table 1).

### Sequence analyses

Sequences were aligned using Clustal*W* in MEGA4 (Tamura et al., 2007) and general sequence statistics were also calculated in MEGA4 (Tamura et al., 2007). jModelTest was used to statistically select the best fit model of nucleotide substitution for data analysis (Guindon and Gascuel, 2003; Felsenstein, 2005; Posada, 2008). The HKY (Hasegawa et al., 1985) model of nucleotide substitution gave the best fit for the data. A phylogenetic maximum-likelihood tree was constructed using RAxML (Stamatakis, 2006). Support values for inferred relationships between taxa were estimated using 1000 bootstrap (BP) replicates. We conducted Bayesian phylogenetic analysis in MrBayes (Huelsenbeck and Ronquist, 2001) using the HKY model (Hasegawa et al., 1985) with inverse gamma-distributed rate variation across sites and a proportion of invariable sites. The analysis was run for 200,000 generations with the sampling frequency set to every 100th generation. The analysis was continued until the standard deviation of split frequencies was below 0.01 after which the parameter values were summarized with a burn-in value corresponding to 25% of the samples used in each analysis. To ensure that the analyses were not trapped in local optima, the data set was run independently five times. Consensus trees were viewed in TREEVIEW (Page, 1996).

### Southern blot analyses

Ten micrograms of genomic DNA prepared from the *Carthamus* leaves was digested with one of the three restriction enzymes, including *Bgl*II, or *Hin*dIII or *Eco*RV overnight and electrophoresed through 1% agarose gel prior to blotting onto a Hybond-N^+^ nylon membrane (Amersham, UK). The filters were probed with α-P^32^ dCTP-labeled safflower *FAD2-1 or FAD2-2 gene specific* DNA fragment. Hybridization was performed in 6 × SSPE, 10% Denhardt's solution, 0.5% SDS, 100 μg/mL denatured salmon sperm DNA overnight at 65°C. After a brief wash in 2 × SSC/0.1% SDS at 50°C, the filter was washed three times in 0.2 × SSC/0.1% SDS at 50°C for 20 min each prior to autoradiography.

### Fatty acid analyses

The oil from mature safflower seeds was expressed onto a filter paper disc and directly methylated in 2 mL of 0.02 M sodium methoxide for 1 h at 90°C, followed by addition of 1.5 mL of hexane and 2 mL of water. After vortexing and phase separation, the upper hexane layer containing the fatty acid methylesters (FAME) was transferred to a new microvial and was analyzed by Agilent 6890N gas chromatography with a 30 m BPX70 column as described by Cao et al. (2013).

## Results

### DNA sequence analyses of *FAD2-1* and *FAD2-2*

The entire 5′ UTR intron of *FAD2-1* was amplified (1199 bp) from all the 22 *Carthamus* accessions, including 17 *C. tinctorius*, 1 *C. palaestinus*, and 4 *C. oxycanthus* accessions, using the primers based on the its conserved flanking regions in the 5′ UTR (Table 1). All of the introns started with GT and ended with AG, consistent with the plant consensus exon/intron boundaries (Simpson and Filipowicz, 1996).

*FAD2-1* intron sequences were found to be highly conserved with a total of 144 variable sites and 4 haplotypes identified across all species. Haplotype 1 was present in *C. palaestinus* and *C. tinctorius* (including PI 401577, the high oleic acid accession); haplotype 2 was unique to the *C. tinctorius* PI 599253, the high oleic acid cultivar S-317; haplotype 3 was exclusive to *C. oxyacantha*; and haplotype 4 was identified in *C. tinctorius*, including the PI 538779, the high linoleic cultivar Centennial.

**Figure 1:**
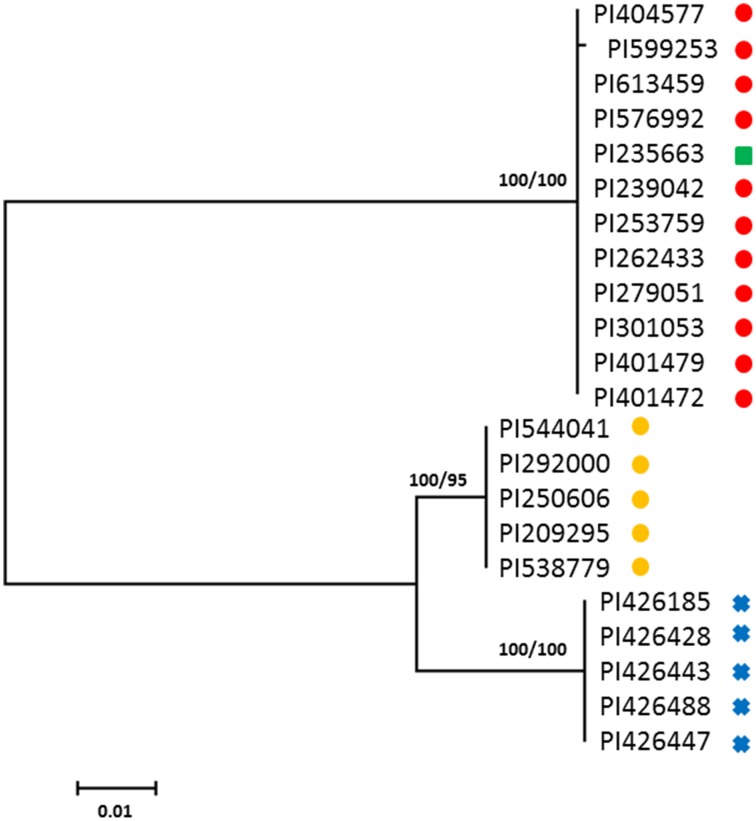
**Phylogenetic maximum-likelihood trees obtained from the analysis of ***FAD2-1*** datasets for three species: ***C. oxyacantha***, ***C. palaestinus***, and ***C. tinctorius*****. Bootstrap values are reported for each node unless less than 70 (RAxML/MrBayes). The scale bar represents the branch length as a measure of substitutions per site. 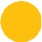, *C. tinctorius* group A; 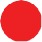, *C. tinctorius* group B; 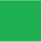, *C. palaestinus*; 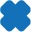, *C. oxyacantha*.

Maximum likelihood analysis of *FAD2-1* sequences shows *C. oxyacantha* is clearly monophyletic (bootstrap = 100%), however, *C. tinctorius* appears to be paraphyletic (Figure 1). Eleven *C. tinctorius FAD2-1* sequences form a strongly supported clade with *C. palaestinus*. The remaining 5 *C. tinctorius FAD2-1* sequences create a separate clade. All of the *C. oxyacantha* group together. Two different *FAD2-1* intron sequences, belonging to the two distinct clades mentioned above, likely with a heterozygous *FAD2-1* locus, were isolated from PI 271070 and it was therefore subsequently removed from the analysis. The two high oleic acid mutants PI 401577 (S-317) and PI 401479, harboring the *ol* allele, were closely associated with *C. palaestinus*, along with 9 other *C. tinctorius* accessions.

Similar to *FAD2-1*, the entire 5′ UTR intron of *FAD2-2* (3178 bp) was amplified from all the 22 *Carthamus* accessions, using the primers based on its conserved flanking regions in the 5′ UTR (Table 1). Again, all of the introns started with GT and ended with AG.

**Figure 2:**
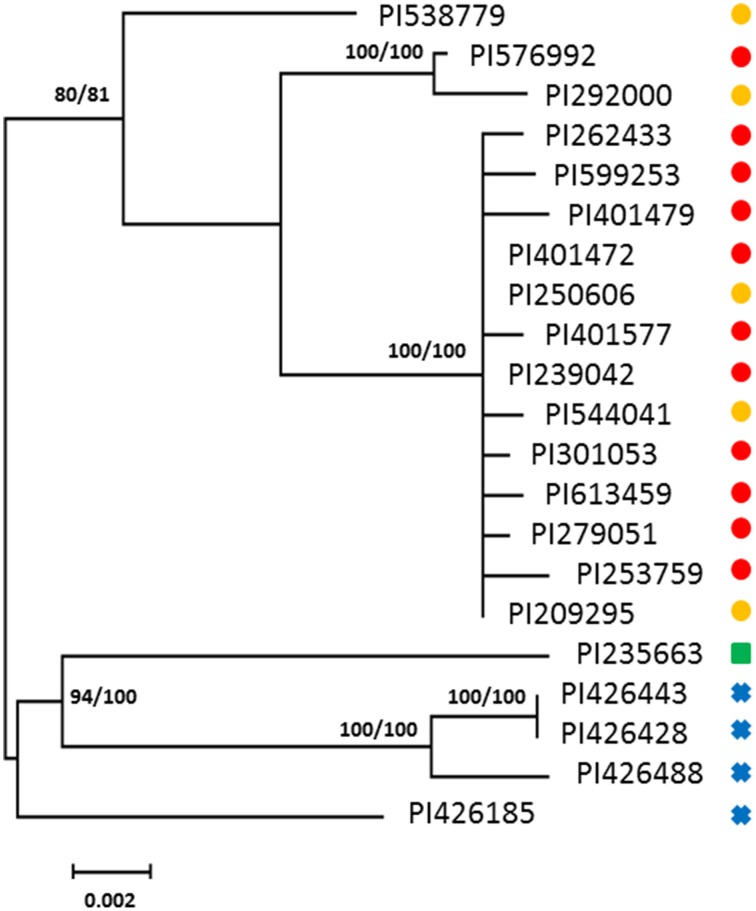
**Phylogenetic maximum-likelihood trees obtained from the analysis of ***FAD2-2*** datasets for three species: ***C. oxyacantha***, ***C. palaestinus***, and ***C. tinctorius*****. Bootstrap values are reported for each node unless less than 70 (RAxML/MrBayes). The scale bar represents the branch length as a measure of substitutions per site. 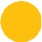, *C. tinctorius* group A; 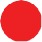, *C. tinctorius* group B; 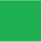, *C. palaestinus*; 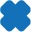, *C. oxyacantha*.

**Figure 3:**
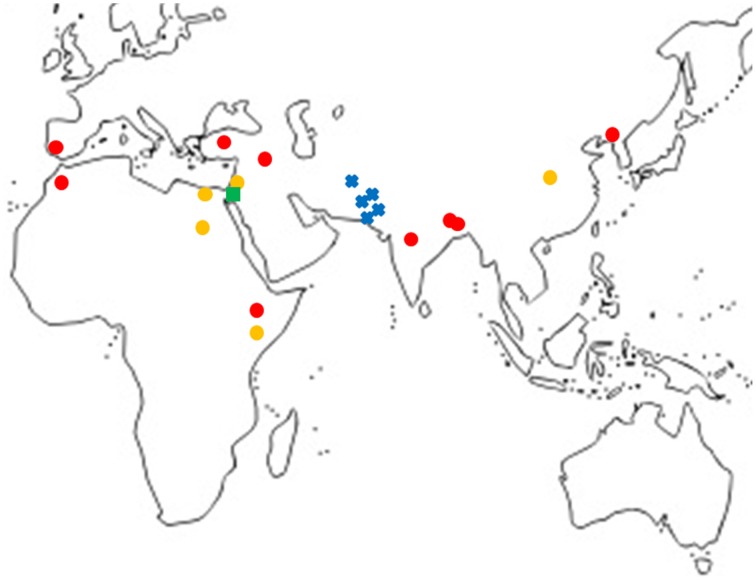
**Map showing the locations of samples grouped according to ***FAD2-1*** sequences excluding cultivars**. 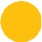, *C. tinctorius* group A; 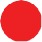, *C. tinctorius* group B; 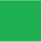, *C. palaestinus*; 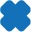, *C. oxyacantha*.

A total of 18 haplotypes of *FAD2-2* were identified with 178 variable sites across all species, including 4 haplotypes for *C. oxycanthus* (*n* = 5); 1 haplotype for *C. palaestinus* (*n* = 1); and 13 haplotypes for *C. tinctorius* (*n* = 16). In phylogenetic analyses all the three species were genetically distinct from one another. In contrast to *FAD2-1, C. oxyacantha* and *G. palaestinus FAD2-2* appeared to be more closely related to each other than to *C. tinctorius* (Figure 2). We have tracked the origin of samples and found that all clades appear to be widespread across the globe, without a distinct distribution pattern (Figure 3).

### Southern blot analyses

**Figure 4:**
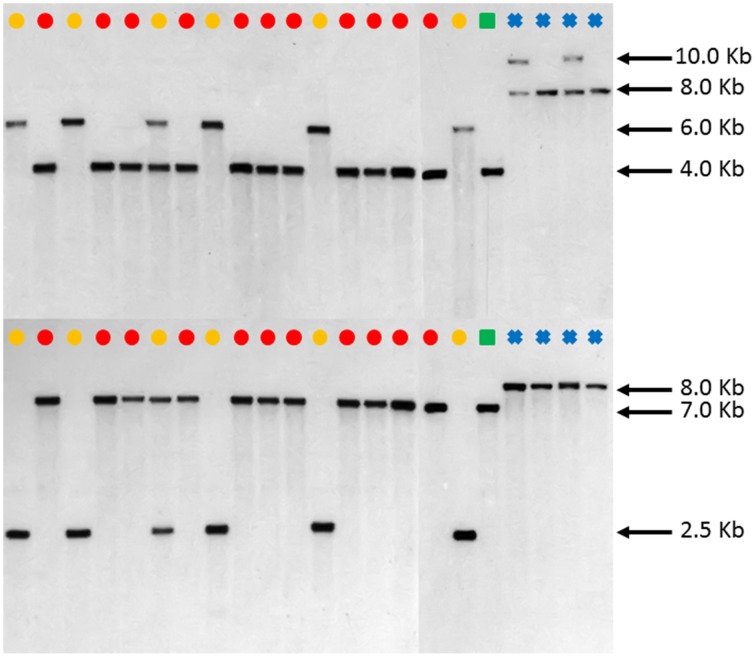
**Southern blot ***FAD2-1*** digestion radiographs (top using enzyme ***Bgl***II, bottom using enzyme ***Eco***RV)**. Samples are in order according to their listing in Table 1 starting with PI 209295 and ending with PI 426488. 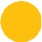, *C. tinctorius* group A; 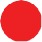, *C. tinctorius* group B; 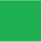, *C. palaestinus*; 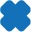, *C. oxyacantha*.

DNA blot analysis of *FAD2-1* using enzymes *Bgl*II and *Eco*RV consistently showed three distinct restriction fragment length polymorphism (RFLP) patterns in *C. tinctorius*. Group 1 consisting of 5 accessions, as labeled by a yellow dot in Figure 4, showed a single band of approximately 6.0 kb by *Bgl*II digestion, and 2.5 kb by *Eco*RV digestion. Group 2 consisting of 11 other accessions, as labeled by Red dots, showed a single band of approximately 4.0 kb in length by *Bgl*II digestion, and 7.0 kb by *Eco*RV digestion. Group three is a single accession, PI 271070 labeled by a yellow dot, showed two bands in either restriction enzyme digestion, combining the RFLP pattern of both group 1 and group 2. This particular plant was likely a heterozygous hybrid between groups 1 and 2 of *C. tinctorius*. Of particular interest, in both *Bgl*II and *Eco*RV digestions, *C. palaestinus* (labeled by a green square) showed the exactly same RFLP pattern as that of the Group 2 in *C. tinctorius*. This is consistent with the sequence analysis of *FAD2-1* intron, which showed higher sequence similarity between group 2 *C. tinctorius* and *C. palaestinus*, distinct from group 1 *C. tinctorius*. In both restriction enzyme digestions, the four *C. oxycanthus* accessions showed distinct patterns from either *C. tinctorius* or *C. palaestinus*.

**Figure 5:**
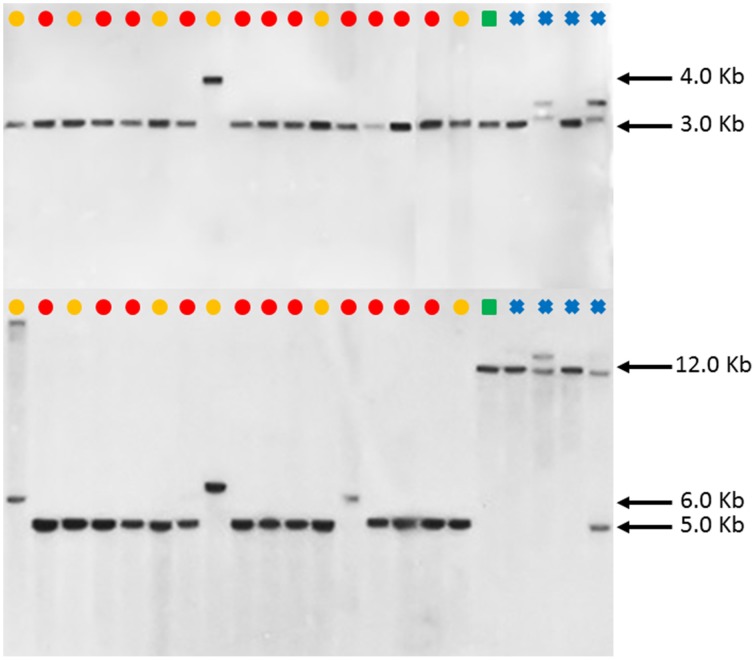
**Southern blot ***FAD2-2*** digestion radiographs (top using enzyme ***Bgl***II, bottom using enzyme ***Hin***dIII)**. Samples are in order according to their listing in Table 1 starting with PI 209295 and ending with PI 426488. 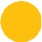, *C. tinctorius* group A; 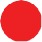, *C. tinctorius* group B; 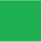, *C. palaestinus*; 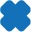, *C. oxyacantha*.

However, such an observation was not made in the *FAD2-2* RFLP patterns as illustrated in Figure 5. Except PI 292000, the other 16 *C. tinctorius* accessions (labeled with yellow or red dots) shared the same RFLP pattern (a band of approximately 3.0 kb) with *C. palaestinus* (labeled with a green square) and two *C. oxycanthus* (labeled with blue crosses) in the *Bgl*II digestion, whereas the *C. palaestius* showed a clear distinction from all the 17 *C. tinctorius* accessions, but shared a band of approximately 12.0 kb with all four *C. oxycanthus* in the *Hin*dIII digestion.

### Fatty acid analyses

The fatty acid composition of the seed oil from each of the 22 accessions of *Carthamus* spp. was analyzed. The majority of samples, except two, contained high levels of linoleic acid, ranging between 71 and 82%, and an oleic acid content of between 8 and 17% (Table 2). The two high oleic acid *C. tinctorius* accessions were PI 599253 containing 74.5 ± 1.4 and PI 401479 containing 80.7 ± 3.2% oleic acid, respectively. Both high oleic accessions also contained low concentrations of linoleic acid, showing a clear precursor/product relationship. PI 599253 is a commercial cultivar developed in the California, USA, by incorporating the *ol* allele originally found in an Indian accession. PI 401479 contains significantly higher level of oleic acid than S-317, indicating that a different mechanism may operate between these two high oleic acid mutants.

**Table 2:** **Fatty acid composition of ***Carthamus*** spp. seed oil**.

**Sample**	**C16:0 Palmitic**	**C18:0 Stearic**	**C18:1 Oleic**	**C18:2 Linoleic**	**C18:3n3 α-Linolenic**	**C20:0**	**C20:1^Δ11^**	**C22:0**
PI 239042	8.6±0.4	2.3±0.1	10.3±0.8	78.0±1.2	0.2±0.0	0.3±0.0	0.2±0.0	0.2±0.0
PI 250606	8.4±0.2	2.2±0.0	10.1±0.2	78.4±0.1	0.2±0.0	0.3±0.0	0.2±0.0	0.2±0.0
PI 253759	8.1±0.6	2.8±0.2	11.3±0.7	76.9±0.8	0.2±0.0	0.3±0.0	0.2±0.0	0.2±0.0
PI 262433	8.4±0.1	2.4±0.1	10.4±0.5	77.9±0.8	0.2±0.0	0.3±0.1	0.2±0.0	0.2±0.0
PI 271070	7.6±0.3	2.5±0.0	11.4±1.0	77.8±0.7	0.2±0.1	0.3±0.0	0.2±0.0	0.2±0.1
PI 279051	7.4±0.4	2.4±0.1	10.5±0.7	78.9±0.6	0.2±0.0	0.3±0.0	0.2±0.0	0.2±0.0
PI 292000	7.7±0.1	2.3±0.2	9.0±0.5	80.1±0.5	0.2±0.0	0.2±0.0	0.2±0.0	0.2±0.0
PI 301053	7.2±0.3	2.8±0.2	13.2±0.7	75.7±1.0	0.3±0.1	0.3±0.0	0.2±0.0	0.2±0.0
PI 401472	7.2±0.5	2.5±0.4	17.6±1.4	71.8±2.6	0.2±0.0	0.3±0.0	0.2±0.0	0.3±0.0
PI 401479	5.9±0.1	1.7±0.2	80.7±3.2	10.5±3.8	0.1±0.0	0.4±0.0	0.4±0.1	0.3±0.0
PI 544041	7.5±0.1	2.9±0.3	11.6±0.5	77.0±0.7	0.2±0.0	0.4±0.0	0.2±0.1	0.2±0.0
PI 576992	6.9±0.0	2.3±0.1	10.1±0.9	79.2±0.8	0.2±0.1	0.4±0.1	0.2±0.1	0.7±0.0
PI 613459	8.3±0.6	1.8±1.1	11.2±0.8	77.8±1.3	0.3±0.0	0.2±0.1	0.2±0.1	0.3±0.1
PI 599253	7.0±0.7	2.6±0.5	74.5±1.4	14.6±1.4	0.1±0.0	0.4±0.1	0.4±0.1	0.4±0.0
PI 401577	8.1±0.4	2.3±0.1	10.3±0.8	78.5±1.2	0.2±0.0	0.3±0.0	0.2±0.0	0.2±0.0
PI 538779	7.9±0.2	2.2±0.0	10.1±0.2	78.9±0.1	0.2±0.0	0.3±0.0	0.2±0.0	0.2±0.0
PI 235663	6.4±0.1	2.7±0.0	8.0±0.1	80.4±0.6	0.2±0.0	0.3±0.0	1.2±0.4	0.1±0.0
PI 426185	6.1±0.2	2.3±0.1	8.5±0.7	81.6±0.8	0.2±0.0	0.4±0.0	0.2±0.1	0.7±0.1
PI 426428	6.4±0.9	1.9±0.2	8.4±0.7	81.9±1.7	0.2±0.0	0.3±0.0	0.3±0.0	0.6±0.1
PI 426443	6.2±0.6	2.5±0.4	8.3±0.6	82.0±0.8	0.2±0.2	0.3±0.0	0.2±0.1	0.4±0.1
PI 426447	6.5±1.1	1.7±0.3	8.7±0.7	81.7±1.7	0.1±0.1	0.3±0.0	0.3±0.0	0.6±0.1
PI 426488	6.9±0.6	2.8±0.0	7.9±0.1	81.1±0.7	0.2±0.0	0.3±0.0	0.2±0.0	0.6±0.1

*Percentages of each compound within a sample are shown with the total adding up to 100% (Mean ± Stdev)*.

## Discussion

In this study we have found that among *FAD2-1* intron sequences of *C. tinctorius* accessions sampled across the world, there is a group aligned closely with that of *C. palaestinus*, while the remaining sequences clearly support the conclusions of Chapman et al. (2009), showing *C. oxyacantha, C. palaestinus*, and *C. tinctorius* are all genetically distinct from one another. Such a division within *C. tinctorius* was not observed in *FAD2-2* intron. We have also presented corroborative evidence showing three distinct RFLP patterns for *FAD2-1* that differed to the RFLP patterns for *FAD2-2*. From these analyses we propose that there was a natural outcrossing event that led to *FAD2-1* gene introgression from *C. palaestinus* into *C. tinctorius*.

Genetic introgression is unidirectional (Martinsen et al., 2001) and occurs as a result of repeated backcrossing of hybrids to the parental species (Stewart et al., 2003). Interspecific gene flow between cultivated and weedy *Carthamus* species (Berville et al., 2005), and other Compositae crops, such as sunflower (Burke et al., 2002), chicory (Kiaer et al., 2007), and lettuce (Hayes and Ryder, 2007) have been reported, fuelling debates about transgene escape in the evolution of super weeds. In this study, we have found clear evidence for the introgression of the *FAD2-1* gene from *C. palaestinus* into more than half of the *C. tinctorius* samples examined. This finding is not unexpected as hybrids between these two species can be readily acquired under field conditions (Ashri and Rudich, 1965; Heaton and Klisiewicz, 1981).

The cultivated safflower is believed to have had a single origin of domestication in the Fertile Crescent region approximately 4000 years ago, and reproductive barriers between crops and wild progenitors appears to be weak (Weiss, 2000). The ability of safflower to intercross and produce fertile offspring with *C. palaestinus* or *C. oxyacanthus* has been well documented (Ashri and Knowles, 1960; Ashri and Rudich, 1965; McPherson et al., 2004). All three species have the same number of chromosomes and the distribution of *C. palaestinus* and *C. oxyacanthus* in the Near East is consistent with safflower originating in this region. In addition, *C. palaestinus* is considered to be the progenitor species of safflower (Chapman and Burke, 2007). It is therefore plausible to suggest that the hybridization between *C. palaestinus* and *C. tinctorius* has occurred. Furthermore, given that *C. palaestinus* is only found in desert areas of Western Iraq, Jordan, and Southern Israel (Knowles, 1976; Smith, 1996), this particular hybridization event is most like to have occurred within this area.

Since its initial domestication from the Fertile Crescent, safflower cultivation has spread to other parts of the world. Initially safflower was classified into phenotypically distinguished seven “centers of similarity” including the Far East, India-Pakistan, the Middle East, Egypt, Sudan, Ethiopia, and Europe (Knowles, 1969; Knowles and Ashri, 1995). The combination of poor reproductive barriers and the opportunity for secondary contact *via* migration may have allowed the introgression of *FAD2-1* from *C. palaestinus* into *C. tinctorius*. In addition, the long-term retention of ancestral polymorphism observed in *FAD2-1* in *C. tinctorius* could be attributable to migrant alleles from occasional interspecific hybridization with *C. palaestinus*. These migrant alleles, represented by *FAD2-1* as described here, may increase the diversity within populations and slow the process of allelic coalescence. A substantial increase in the sample size for *C. palaestinus* is clearly needed to more accurately understand the introgression event of *FAD2-1*. Additional information on the conditions, such as climatic variation, which may have enabled spatial and temporal sympatry of the two species and subsequently their hybridization in the Fertile Crescent, would also improve our understanding of this event.

It is likely that the introgression *FAD2-1* from *C. palaestinus* to *C. tinctorius* was a single event that spread to more than half of the safflower samples chosen randomly from around the world. If the introgression had been extensive and dating further back in time, we expect that the *C. palaestinus FAD2-1* gene would have been more evenly distributed among all the *C. tinctorius* individuals and harder to recognize as an introgressed gene. The mutant allele conferring the high oleic acid trait in *C. tinctorius* appears to have a more recent origin than the introgression event, by deriving from a single nucleotide deletion in the coding region of *FAD2-1* following its introgression into *C. tinctorius*.

A typical oleic acid profile conferred by the *ol* allele is about 74% as shown here in PI 599253 (Table 2), a cultivar derived from the original high oleic germplasm identified by Knowles and Hill (1964). In this study we have included another high oleic acid *C. tinctorius* accession, PI 401479, which accumulates more than 80% oleic acid in its total fatty acids, clearly above the upper-margin of *ol* mutant (Table 2). This very high oleic acid germplasm was identified by Fernández-Martinez et al. (1993) and genetically characterized by Hamdan et al. (2009, 2012) who demonstrated that the additional oleic acid content (compared to that of PI 599253) was a result of the combined effects of the recessive *ol* allele and a number of other modifying genes with a positive effect on oleic acid content, as previously suggested by Knowles (1972). In confirmation of this hypothesis, we found that the *FAD2-1* sequences of both PI 599253 and PI 401479 are identical and contain the same genetic mutation of a single nucleotide deletion linked to the high oleic acid trait (data not presented).

Genetic resources remain critically important for the development and improvement of safflower cultivars and germplasm. The identification and characterization of the genetic diversity of *FAD2-1* enables the exploitation of the high oleic acid trait by safflower breeders in an efficient manner. Further, the development of molecular markers from expressed sequences from genes of importance, such as *FAD2-1* in this case, should aid plant breeders in reducing the population size and number of generations required for the development of new high oleic acid varieties by using marker-assisted selection. In this context it is pivotal to understand the genetic origin and variation of *FAD2-1* alleles in safflower population.

The relative level of oleic/linoleic acid in *Carthamus* seed oil controlled by the expression of *FAD2-1* is known as a neutral trait that does not offer any evolutionary advantages. Nevertheless, the case study reported here will contribute to a growing body of literature showing that crop origins are genetically more complex than once thought. Beyond the current study, the whole genome sequencing approach may provide detailed information on genetic content and the origins of the introgressed regions through comparison to genomes of *C. tinctorius* and *C. palaestinus*. For instance, in a recent study with next-generation sequencing, significant introgression of genes from wild species to cultivated crops, and interspecific gene flow has left its imprint in the genome of several Compositae crops and their wild relatives (Hodgins et al., 2014). Through the current case study with *FAD2-1*, it is tempting to speculate that the diversity of safflower germplasm is not only complex in genetic structure but also dynamic with constantly evolving entities. Reticulate evolution, facilitated by introgression, may have also influenced the observed complexities among *Carthamus* species.

Furthermore, wild progenitor species and feral forms of domesticated species have been reservoirs of genetic variations that could be beneficial to crops, such as disease resistance or increased stress tolerance (Tanksley and McCouch, 1997; McCouch, 2004; Fernie et al., 2006). For example, bacterial resistance gene *Pto*, which was introgressed from the wild tomato species, *S. pimpinellifolium*, in the 1930's, and later positionally cloned (Martin et al., 1993; Pedley and Martin, 2003). Despite the current availability of transgenic approaches allowing tapping of tertiary gene pools from distant taxa, primary and secondary gene pools from the same and related species are still the most important sources of genetic variation for plant breeders.

### Conflict of interest statement

The authors declare that the research was conducted in the absence of any commercial or financial relationships that could be construed as a potential conflict of interest.
